# A strategy for addicting transgene-free bacteria to synthetic modified metabolites

**DOI:** 10.3389/fmicb.2023.1086094

**Published:** 2023-02-10

**Authors:** Yusuke Kato

**Affiliations:** Institute of Agrobiological Sciences, National Agriculture and Food Research Organization (NARO), Tsukuba, Japan

**Keywords:** biological containment, synthetic nucleotides, synthetic auxotrophy, thymineless death, cartagena protocol on biosafety

## Abstract

Biological containment is a safeguard technology to prevent uncontrolled proliferation of “useful but dangerous” microbes. Addiction to synthetic chemicals is ideal for biological containment, but this currently requires introduction of transgenes containing synthetic genetic elements for which environmental diffusion has to be prevented. Here, I designed a strategy for addicting transgene-free bacteria to synthetic modified metabolites, in which the target organism that can neither produce an essential metabolite nor use the extracellularly supplied metabolite, is rescued by a synthetic derivative that is taken up from a medium and converted into the metabolite in the cell. Because design of the synthetic modified metabolite is the key technology, our strategy differs distinctly from conventional biological containment, which mainly depends on genetic manipulation of the target microorganisms. Our strategy is particularly promising for containment of non-genetically modified organisms such as pathogens and live vaccines.

## Introduction

Microorganisms contribute to enrichment of human life (Demain and Adrio, 2008; Alexandra and Branco, 2017), but many useful species can also be dangerous. These include genetically modified microorganisms for which safety has yet to be confirmed, pathogens used in therapeutic research and live vaccine development, and environmentally invasive microorganisms with high adaptability (Berg et al., 1975; Berns, 2014; Marbuah et al., 2014). These “useful but dangerous” microorganisms need to be contained to prevent uncontrolled proliferation in the open environment.

Physical containment such as safety cabinets, air filtration, and autoclave sterilization is currently used for microbial containment, based on physical barriers to prevent microorganisms escaping into the environment (World Health Organization, 2020). However, equipment failure or human error limits effective physical containment, and leakage due to accidents or disasters always a possibility. Furthermore, environmental pollutants such as toxic chemicals and radioactive materials decay over time, whereas microorganisms can proliferate autonomously and spread widely in the environment after a leak. These characteristics suggest that containment of microorganisms must be perfect, and this is inconsistent with physical containment.

Microbial containment can also be achieved through biological containment (Torres et al., 2016; Lee et al., 2018; Whitford et al., 2018; Stirling and Silver, 2020), which relies on genetic programming of a target organism to survive only under human control and die in the natural environment. Biological containment uses an autonomous system that is genetically embedded in the microorganism, which eliminates concerns of human error or equipment failure, as may occur in physical containment. Thus, biological containment may allow perfect microbial containment, and achievement of this goal without physical containment may expand applications of “useful but dangerous” microorganisms to use in open environments.

With this background, I considered how ideal biological containment might logically be achieved. The target microorganism should survive only when a survival-permissive factor is provided. In most biological containment systems proposed to date, a specific chemical has been used as this factor, and appropriate selection of the survival-permissive factor is a particularly important issue. Natural chemicals used in organisms, such as essential nutrients and metabolites, were used as survival-permissive factors in early biological containment systems because these systems can be easily constructed by disrupting genes in pathways that produce these chemicals or by constructing synthetic genetic circuits that control life and death using natural genetic elements regulated by the chemicals (Ramos et al., 2011). Natural chemicals that are rare in the environment are usually selected, but the target organisms may still have access to these chemicals. In fact, there have been many reports in which biological containment has failed due to the existence of unexpected environmental niches and resources providing the rare natural chemicals (Ramos et al., 2011), as if it were a fable about the dinosaurs in Jurassic Park (Kato, 2015). By using multiple layers of biological containment, the incidence of such failures can be reduced, but not to zero (Lee et al., 2018).

These findings suggest that synthetic chemicals or nutrients/metabolites that are not in the natural environment are ideal as survival-permissive factors. Several biological containment methods using addiction to synthetic chemicals have recently been reported, including unnatural amino acid-dependent production or functionalization of essential proteins, such as those encoded in genomes, and antitoxins in artificially introduced toxin-antitoxin systems (Kato, 2015; Mandell et al., 2015; Rovner et al., 2015; Gan et al., 2018; Kato, 2019). However, artificially modified genes are needed because the gene products that interact with the synthetic chemicals do not usually exist in nature. Therefore, if a synthetic chemical is selected as a survival-permissive factor, the target microorganism has to be genetically modified to carry transgenes that are distinct from the natural genes. For such organisms, containment of transgenes is a serious problem due to the risk of incorporation of artificial genetic elements into the natural gene pool and resultant unpredictable effects on ecosystems (Stirling and Silver, 2020). Even if biological containment works ideally and kills all the target organisms, environmental release of transgenes from dead cells may still occur. Use of genetically modified organisms is also subject to strict legal regulations (Eggers and Mackenzie, 2000). Consequently, unnatural genetic elements should not be used in biological containment. A transgene-free system is preferable, although degrading transgenes using non-specific or CRISPR nucleases is also a promising solution (Ramos et al., 2011; Caliando and Voigt, 2015). These considerations are particularly important for containment of non-genetically modified organisms such as live vaccines.

**Figure 1:**
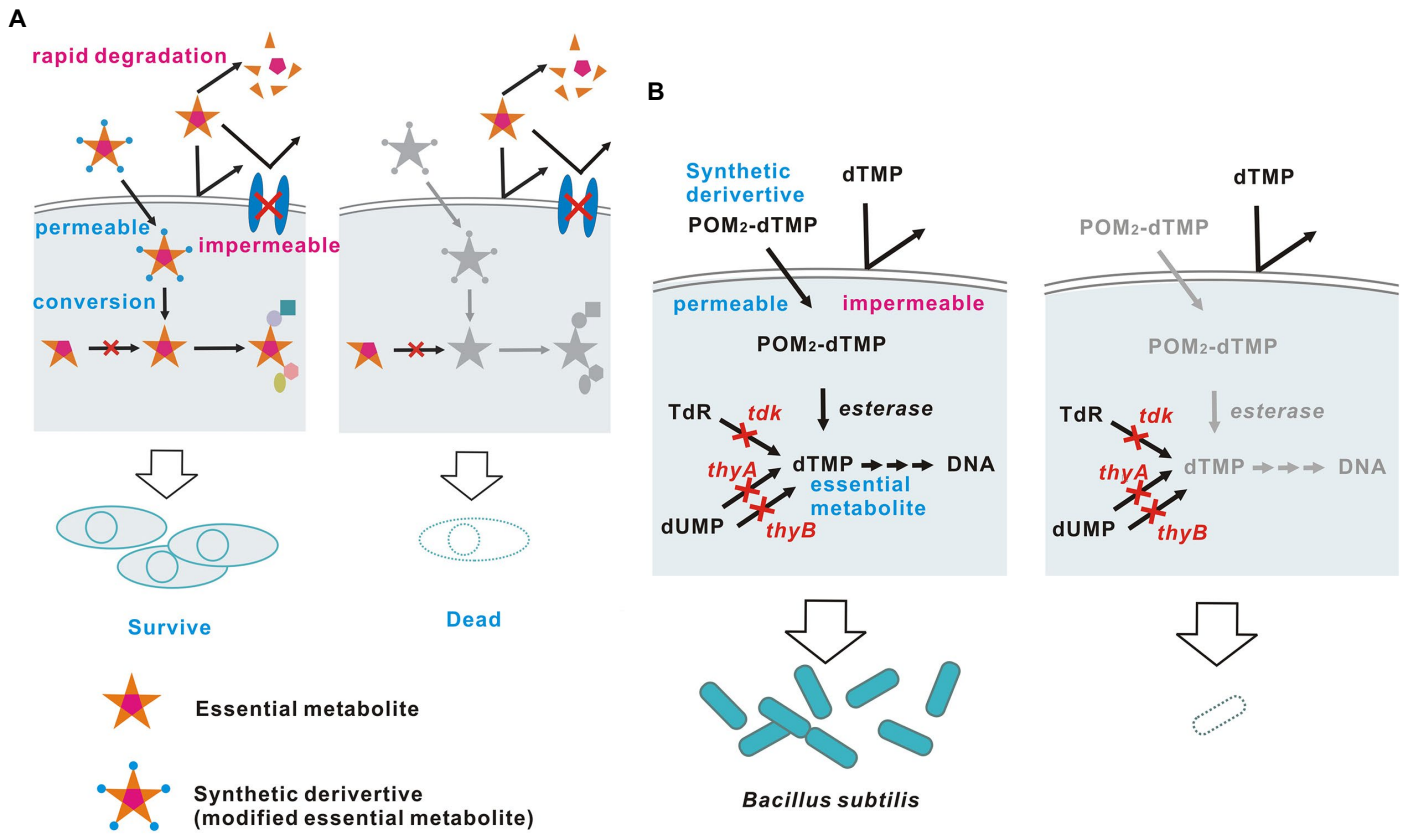
Theoretically ideal biological containment. **(A)** Schematic diagram and **(B)** The embodied containment system. The membrane-permeable dTMP derivative POM_2_-dTMP is used as an example of a synthetic survival-permissive factor.

A biological containment strategy that satisfies the above conditions through addiction of organisms to synthetic chemicals or nutrients/metabolites without using transgenes is ideal in principle, but has not been achieved to date. I examined how this principle could be achieved (Figure 1A) and concluded that systems should satisfy the following three conditions. First, a nutrient/metabolite that is essential for survival is selected as a survival-permissive factor. This factor should be unavailable to the target organism when supplied extracellularly, due to membrane impermeability, absence of transporters, or rapid degradation. Second, the target organism must lose all pathways for synthesis and transport of the survival-permissive factor by gene disruption. The gene disruption should be accomplished without use of a transgene, using spontaneous mutation, chemically or physically induced mutation, or genome editing that leaves no trace. The target organism generated in this manner is not a genetically modified organism for legal and regulatory purposes in many countries (Petersen et al., 2022). Third, the survival-permissive factor must be modified into a form that can be used when supplied extracellularly, and the modified factor must not exist in nature. Consequently, the target organism can survive only with a supply of this factor. In this study, I designed a biological containment system that embodies these theoretically ideal principles in bacteria and I provide a proof of concept.

## Results

### Embodiment of theoretically ideal containment

First, 2'-deoxythymidine 5'-monophosphate (dTMP) (Figure 1B) was chosen as a suitable metabolite to serve as a survival-permissive factor. Since 2'-deoxythymidine 5'-triphosphate (dTTP), which is essential for DNA synthesis, is produced *via* a single pathway that uses dTMP as a precursor, dTMP is an essential metabolite for most organisms (Ahmad et al., 1998). Also, nucleoside phosphates including dTMP cannot pass through biological membranes without a carrier protein due to their large size and charge (Winkler and Neuhaus, 1999; Di Noia et al., 2014; Alonzo et al., 2018). Thus, most bacterial species cannot utilize dTMP supplied from outside the cell. In addition, dTMP starvation causes cell death (thymineless death), which is advantageous for biological containment because other nutrient starvation merely stops growth (Ahmad et al., 1998; Steidler et al., 2003).

Next, I examined ways to enable dTMP non-producing bacteria to acquire dTMP. dTMP must be modified to be taken up by cells. Many modifications that confer membrane permeability to nucleoside phosphates have been reported for intracellular delivery of prodrugs used as antiviral and anticancer agents (Pradere et al., 2014). A common strategy is masking to neutralize the negative charge of the phosphate, thereby increasing cell permeability. Once inside the cell, the mask is enzymatically removed to release a free nucleoside phosphate, as described below in detail.

Finally, the well-characterized Gram-positive bacterium *Bacillus subtilis* was selected as a suitable microorganism to test this biological containment strategy, for the following five reasons. First, thymineless death in this bacterium has been widely studied since the 1960s and the physiological responses and genes involved are well characterized (Farmer and Rotham, 1965; Ahmad et al., 1998). Second, easy gene disruption procedures have been established, and our planned biological containment requires that all dTMP synthesis pathways in the bacterium be blocked by genetic disruption. In *B. subtilis*, genes can be easily disrupted by homologous recombination using the competence to uptake intact DNA directly from outside the cell (Koo et al., 2017). Third, large molecules such as dTMP derivatives can easily access the cell membrane, as Gram-positive bacteria lack an outer membrane (Nikaido, 2003). Fourth, most bacteria, including *B. subtilis*, do not have transporters for uptake of nucleoside phosphates from outside the cell (Winkler and Neuhaus, 1999). Therefore, *B. subtilis* cannot directly utilize extracellular dTMP derived from other viable cells, corpses or degraded dTMP derivatives. Fifth, *B. subtilis* has high intracellular nonspecific esterase activity, which may convert hydrophobic dTMP phosphate esters into free dTMP; in contrast, *Escherichia coli,* another well-known model bacterium, has only low activity (Morono et al., 2004; Antonczak et al., 2009). Thus, a biological containment system was constructed in dTMP non-producing *B. subtilis* using a synthetic dTMP derivative as a membrane-permeable survival-permissive factor that is converted to free dTMP intracellularly by esterases.

### Construction of a dTMP non-producing *B. subtilis* strain

A target *B. subtilis* strain was constructed in which all pathways for synthesis of dTMP were blocked. *B. subtilis* has two such pathways: a *de novo* pathway for conversion of 2'-deoxyuridine 5'-monophosphate (dUMP) to dTMP catalyzed by the thymidylate synthetases ThyA and ThyB; and a salvage pathway for conversion of thymidine to dTMP catalyzed by the thymidine kinase Tdk (Ahmad et al., 1998, Figure 1B). *B. subtilis* can survive and proliferate with ThyA, ThyB or Tdk, indicating that all three genes must lose their function to block production of dTMP in *B. subtilis* completely. Notably, ThyA is thermostable, while ThyB is not (Neuhard et al., 1978, Figure 2A). For experimental convenience, I generated a strain that lost dTMP synthesis in a temperature-sensitive manner by disrupting only *tdk* and *thyA,* and maintaining *thyB* intact. This strain is expected to grow at the permissive temperature, 37°C, but die due to thymineless death at a high temperature, 46°C, because ThyB is inactivated at 46°C.

**Figure. 2:**
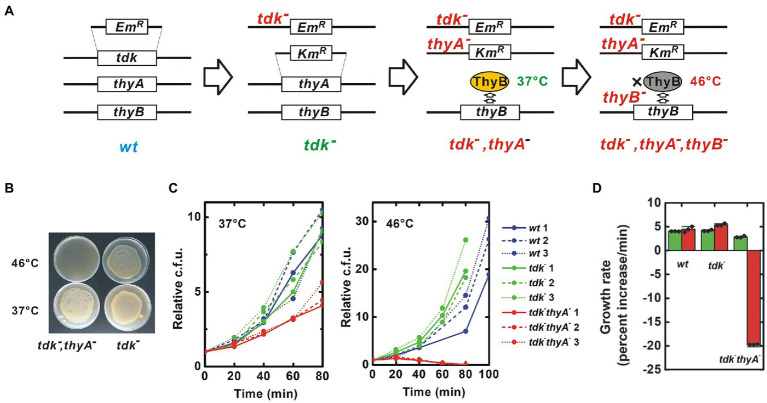
Construction of a thermosensitive dTMP-nonproducing *B. subtilis* strain. **(A)** Procedure for construction of mutant strains. Using natural competence, the ORF of the target gene was substituted with an antibiotic resistance gene cassette. ThyB is a thermosensitive enzyme that is inactivated at 46°C. *Em^R^* and *Km^R^* are erythromycin and kanamycin resistance gene cassettes, respectively, **(B)** Thermosensitive lethal phenotype of the *tdk^−^ thyA^−^* strain. The *tdk^−^ thyA^−^* bacteria were inoculated onto solid medium and incubated overnight at the indicated temperatures. Uneven colony distribution is an artificial phenomenon caused by uneven inoculation, **(C)** Growth curve. Red, green and blue lines indicate *tdk^−^ thyA^−^*, *tdk^−^* and wild-type growth, respectively. The number of viable bacteria is shown relative to the value at Time = 0, and **(D)** The growth rate was calculated from the growth curve shown in **(C)** and indicated as percentage increase of cfu per minute. Green and red column represent the results at 37°C and 46°C, respectively. Negative growth indicates a decrease in the number of viable cells. Values are mean ± sd of three biological replicates.

Although the target genes can be disrupted without transgenes, here I used antibiotic resistance marker genes for convenience of screening and maintenance (Figure 2A; Supplementary Figure 1). A *tdk^−^* strain generated by substitution with the erythromycin resistance gene cassette was obtained from the National BioResource Project (NBRP)-Prokaryote *B. subtilis* (Yamazaki et al., 2010). Disruption of *thyA* by substitution with the kanamycin resistance gene cassette was performed on this strain (Koo et al., 2017; Supplementary Figure 2). I isolated strains resistant to both erythromycin and kanamycin that died at 46°C (Figure 2B). PCR confirmed substitution of *thyA* with the kanamycin resistance gene casette (Supplementary Figure 3). Finally, the genome was sequenced to confirm the *tdk^−^ thyA^−^* double gene disruption. Substitutions of *tdk* and *thyA* with the erythromycin and kanamycin resistance gene cassettes, respectively, were confirmed (Supplementary Figures 4, 5). Several small mutations were also detected, but no clear harmful mutations were predicted (Supplementary Figure 6).

**Figure 3:**
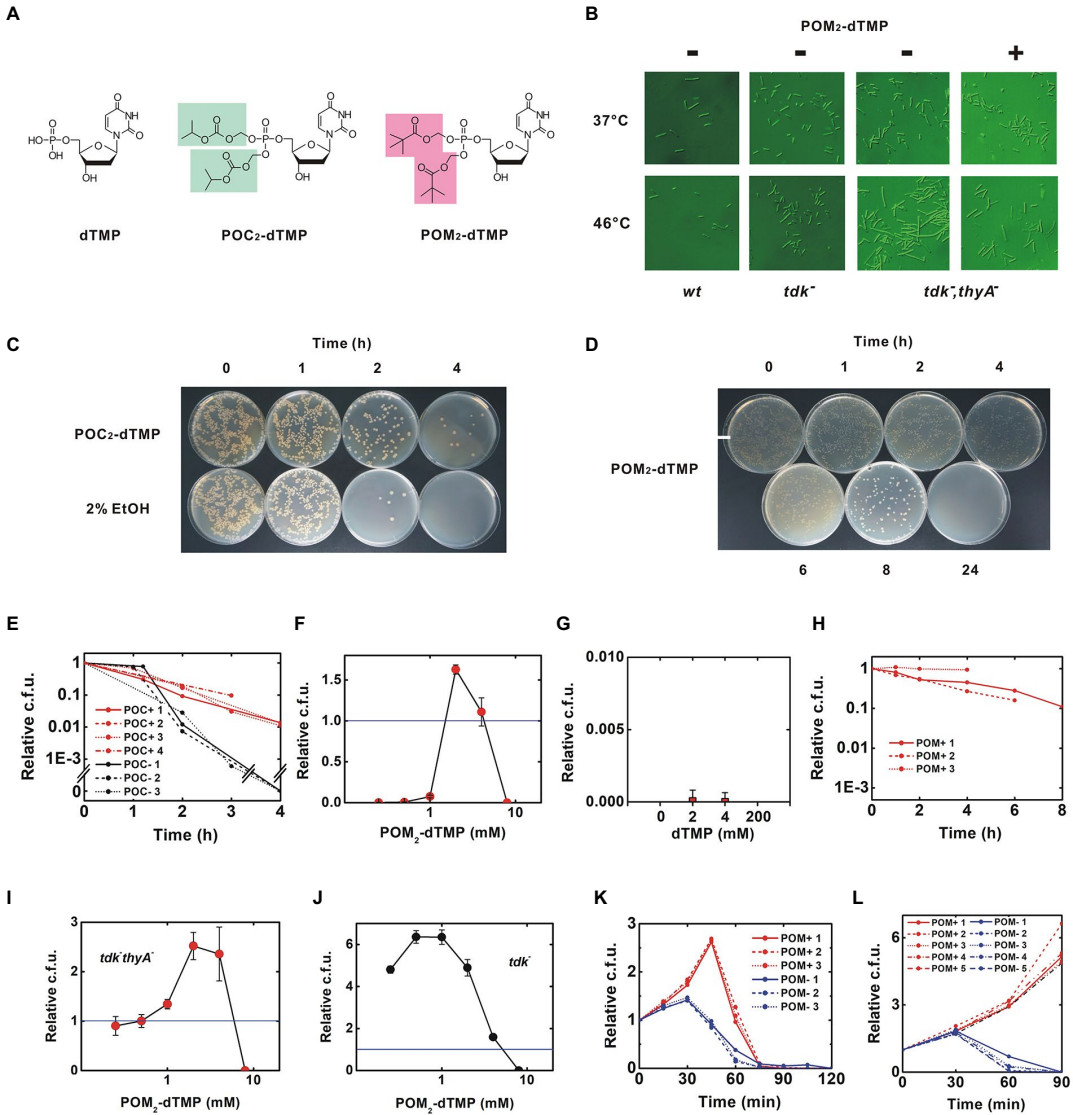
Rescue of the thermosensitive dTMP-nonproducing strain by membrane-permeable synthetic dTMP derivatives. **(A)** Structures of dTMP derivatives. Protective groups added as phosphate esters are highlighted, **(B)** Morphology of *B. subtilis*. After incubation at the indicated temperature for 90 min, the morphology of the bacteria was photographed using a Nomarski differential interference microscope. The calibration bar indicates 50 μm. This experiment was performed in the presence of 0.375% DMSO, **(C–H)** were performed in the presence of 2% ethanol, **(C)** Inhibition of thymineless death by 8 mM POC_2_-dTMP, **(D)** Time course for viability of the *tdk^−^ thyA^−^* strain at 46°C. Red and black lines indicate results in the presence of 8 mM POC_2_-dTMP and solvent control (2% ethanol), respectively. Each trial is presented in a different linetype, **(E)** Inhibition of thymineless death by 4 mM POM_2_-dTMP, **(C,E)** Uneven colony distribution is an artificial phenomenon caused by uneven inoculation. See also Materials and Methods, **(F,G)** Dose-effect relationship for POM_2_-dTMP and dTMP. The *tdk^−^ thyA^−^* strain was incubated at 46°C for 4 h and then the viability was determined. Values are mean ± sd of three biological replicates, **(H)** Effect of POM_2_-dTMP on delaying thymineless death. The *tdk^−^ thyA^−^* strain was incubated at 46°C in the presence of 4 mM POM_2_-dTMP, **(I–L)** were performed in the presence of <1% DMSO. **(I,J)** Dose-effect curves of POM_2_-dTMP on *tdk^−^ thyA^−^* and *tdk^−^* strains. Viable cells were counted after 45 min of incubation at 46°C. Values are mean ± sd of three biological replicates, **(K)** Time course for viability of the *tdk^−^ thyA^−^* strain at 46°C. Red lines indicate experiments in which 3 mM POM_2_-dTMP was supplied at T = 0 without additional supplementation. Blue lines indicate the solvent control (0.375% DMSO), and **(L)** Sustainable proliferation of the *tdk^−^ thyA^−^* strain by continuous supply of POM_2_-dTMP. Fresh POM_2_-dTMP was supplemented every 30 min. For detailed procedures, see Methods.

The *tdk^−^ thyA^−^* strain grew at 37°C due to *de novo* synthesis of dTMP by intact *thyB*, albeit at a reduced growth rate (*p* < 0.003 both to wild-type and *tdk^−^* strain) (Figures 2C,D). In contrast, the *tdk^−^ thyA^−^* strain died after 30–45 min at 46°C, at which ThyB was inactivated, although the wild-type and parental *tdk^−^* strains proliferated normally. The morphology of *tdk^−^ thyA^−^* cells was indistinguishable from that of wild-type and *tdk^−^* cells at 37°C, but an elongated form and filamentation seen in thymineless death occurred at 46°C (Figure 3B). A decrease in dTMP and accumulation of dATP and dUMP were also detected, as seen during thymidylate synthetase inactivation, suggesting that the *tdk^−^ thyA^−^* strain died at 46°C due to thymineless death (Ahmad et al., 1998; Kwon et al., 2010; Supplementary Figure 7). Therefore, the *tdk^−^ thyA^−^* strain was concluded to be a temperature-sensitive *tdk^−^ thyA^−^ thyB^−^* triple mutant.

In a test of the escaper frequency for thymineless death at 46°C (Supplementary Figure 8), no survivors were detected at densities up to 10^6^ cells/plate, but many colonies appeared in plating at higher densities. Individually isolated and recultured survivors died at 46°C, as for the original *tdk^−^ thyA^−^* strain, suggesting that these were not escapers with mutations restoring dTMP synthesis. Similar pseudo-escpaer emergence only in high-density inoculation has been reported previously (Stirling and Silver, 2020). Thus, the escaper frequency was estimated to be under detection limit (10^−6^).

### Rescue with synthetic dTMP derivatives

Membrane-permeable dTMP derivatives were synthesized by esterifying the phosphate with a highly hydrophobic group (Figure 3A, see Methods for details). Isopropyloxymethyl carbonate (POC) and pivaloyloxymethyl (POM) groups were used, based on their previous use in several anticancer and antiviral prodrugs (Pradere et al., 2014). The dTMP derivatives, POC_2_- and POM_2_-dTMP, are likely to permeate the cell membrane without transporters and to be degraded by nonspecific esterases abundant in *B. subtilis*, yielding free dTMP in the cells (Figure 1B). POC and POM both produce degradation products when eliminated from dTMP, but those of POC are less toxic. However, POM is more stable than POC and is less sensitive to changes in pH (Yuan et al., 2001; Wiemer and Wiemer, 2014). For example, POM_2_-ddUMP is stable under pH 1.0–9.0 with halflives ranging from 1 day to 7 days although a rapid degradation occurs in solutions containing esterase such as human plasma (Khan et al., 2005).

These dTMP derivatives could be dissolved in 50% ethanol and 100% dimethyl sulfoxide (DMSO) at concentrations >100 mM. Initially, ethanol was selected as the solvent (Freed et al., 1989). Under our experimental conditions, final 2% ethanol was contained in the bacterial culture, but it was later shown that growth of *B. subtilis* was significantly inhibited and completely arrested in the co-presence of 4 mM POM_2_-dTMP (Supplementary Figure 9). This growth arrest is due to the SigB-induced transition to the stationary phase (Boylan et al., 1993) and bacteria in this phase are expected to survive longer because they are resistant to thymineless death (Ahmad et al., 1998). I first tested the effect of the less toxic POC_2_-dTMP on delaying thymineless death in the presence of ethanol. In a preliminary study, a delay was observed for POC_2_-dTMP at >2.7 mM. Time course experiments clearly showed that 8 mM POC_2_-dTMP delayed thymineless death at 46°C (*p* < 0.001 for the viability at 4 h) (Figures 3C,D). Next, the more stable POM_2_-dTMP was tested. As observed for POC_2_-dTMP, thymineless death was inhibited at concentrations above a few mM, but the bacteria were killed at 8 mM (Figures 3E,F). No effect was observed for free dTMP supplied in the medium (Figure 3G). Since the mean effect of POM_2_-dTMP was the higher of the two derivatives although statistical significance was not evidenced (the mean viability at 4 h was 0.553 ± 0.347 and 0.0127 ± 0.002 for 4 mM POM_2_-dTMP and 8 mM POC_2_-dTMP, respectively), POM_2_-dTMP was chosen for further analysis (Figures 3D,H).

In contrast to ethanol, <4% DMSO does not inhibit growth of *B. subtilis*, so growth rescue was attempted with POM_2_-dTMP in DMSO (Supplementary Figure 9). More than twofold growth was first observed with POM_2_-dTMP in DMSO (Figure 3I). Similarly to the solution in ethanol, only a narrow concentration range (2–4 mM) was effective, as 8 mM POM_2_-dTMP killed the bacteria. Growth inhibition at 4 mM and sterilization at 8 mM were also observed in the parental strain *tdk^−^*, suggesting toxicity of POM_2_-dTMP at high concentrations (Figure 3J). Growth rescue by 3 mM POM_2_-dTMP persisted for 30–60 min, followed by a rapid decrease in viable counts, presumably due to degradation and consumption reducing POM_2_-dTMP below the effective concentration (Figure 3K). Therefore, fresh medium containing POM_2_-dTMP was supplied every 30 min to maintain the required concentration, and this resulted in sustained growth (Figure 3L). Bacteria exhibited elongated morphology, as seen in the absence of POM_2_-dTMP, but filamentation was only rarely observed (Figure 3B). In addition, the unusual accumulation of dATP and dUMP was resolved, and the dTMP level recovered (Supplementary Figure 7). These results show that feeding with POM_2_-dTMP can rescue the survival and growth of the *B. subtilis tdk^−^ thyA^−^ thyB^−^* triple mutant strain that cannot produce dTMP.

## Discussion

The above results show that we can make bacteria addicted to synthetic modified metabolites independent from transgene. Although we used transgenes as selection markers for experimental convenience, the bacterial strain may be generated without transgenes as shown in Supplementary Figure 1. In addition, we also used a *ts* phenotype of *thyB* because of expensive and difiicult-to-control POM_2_-dTMP as described below, but a loss-of-function mutant can be applied in principal. This strategy, thus, provides a way to addict transgene-free bacteria to synthetic modified nutrients/metabolites.

Since dTMP is an essential metabolite in almost all organisms, the POM_2_-dTMP addiction is theoretically applicable to other microbes. However, physical barriers that interfere with access to the cell membrane, such as the outer membrane of Gram-negative bacteria, or intracellular and extracellular esterase activity may affect the availability of the POM_2_-dTMP addiction (Nikaido, 2003; Antonczak et al., 2009). In addition, several factors that reduce robustness are of concern. The POM_2_-dTMP addiction is an auxotroph-based containment system which needs disrupting genes involved in the dTMP synthesis pathway. As seen in other auxotroph, acquiring those genes through horizontal gene transfer may fail the POM_2_-dTMP addiction (Stirling and Silver, 2020). Although it is difficult to completely prevent all pathways of the horizontal gene transfer, suppression of the transfer including deletion of genes involved in natural competence can reduce the gene acquisition (Clark et al., 2018). To best of my knowledge, membrane permeable nucleotide phosphates such as POM_2_-dTMP have not been reported as natural products. However, it is impossible to prove that such molecules never exist in nature or in living organisms, which would be “devil’s proof.” To ensure the availability of the POM_2_-dTMP addiction, it is recommended to check beforehand whether the target microbes survive without POM_2_-dTMP in the environment where it will be used. *B. subtilis* forms spores that are resistant to various stresses, including desiccation, freezing, heats, toxic chemicals, high pressures, UV and γ-radiation (Setlow, 2014). A previous study on the biological containment of *B. subtilis* have reported that thymineless death is also effective for spore containment (Hosseini et al., 2018), suggesting that the spore formation would not affect the robustness of the POM_2_-dTMP addiction.

Several barriers still remain to the practical application of POM_2_-dTMP addiction. Since POM_2_-dTMP need to be kept within a narrow concentration range, a system that allows for concentration monitoring and continuous medium exchange is necessary. In addition, POM_2_-dTMP is not commercially available at this time. POM_2_-dTMP, however, can be synthesized in a relatively simple process as described in M&M, allowing for lower costs in industrial production.

The *tdk^−^ thyA^−^* strain grew at a reduced growth rate at 37°C. In some previous papers, the growth rate of *thyA^−^* strain has been reported to be almost equally maintained as seen in *wt* (Neuhard et al., 1978; Hosseini et al., 2018). Although the deletion of *tdk* causes some metabolic changes, the cause of the reduced growth rate remains to be elucidated (Li et al., 2016).

To date, biological containment has focused on genetic programs to be incorporated into target microorganisms. Our containment strategy is distinct, in that the key technology lies in the molecular design of synthetic modified nutrients/metabolites, rather than in the genetic programs. Here, a hydrophobic protecting group, which was designed to be eliminated by intracellular enzymes, was added to confer membrane permeability to dTMP, which has been reported to be unable to permeate from outside to inside the cell. More complex and precisely controlled containment that integrates information is possible by adding conditions for elimination of protecting groups dependent on environmental signals such as light, temperature, and chemical co-factors (Bardhan and Deiters, 2019; Larroque-Lombard et al., 2021). This strategy is not limited to dTMP, but could be applied to other essential nutrients and metabolites that are difficult to uptake from outside cells. Loss-of-function mutations in transporters, such as chemically induced or footprint-free genome editing mediated large-deletion mutants, may greatly increase the number of candidate survival permissive factors.

In contrast to complex design of synthetic modified nutrients/metabolites to serve as these factors, simple disruption of a few genes in the target microorganisms should suffice. Such target organisms can be generated without using transgenes and thus without concern for the negative genetic and evolutionary effects caused by transfer of the transgenes to organisms in the environment. In addition, transgene-free target microorganisms are unlikely to be subjected to strict legal regulation in many countries. Thus, our containment strategy may be particularly promising for genetically unmodified target organisms, including live vaccines.

## Materials and methods

### Bacterial culture

LB medium (1% BactoTriptone, 0.5% Bacto Yeast Extract, 1% NaCl) was used to culture all *B. subtilis* strains. Antibiotics (erythromycin 1 mg/l or kanamycin 25 mg/l) were added as needed. Liquid culture was performed in an air incubator with shaking (200 rpm). For preparation of solid medium, 2% agar was added. Bacteria were incubated at 37°C unless otherwise noted.

### Construction of a temperature-sensitive *tdk^−^ thyA^−^ thyB^−^* triple mutant strain

A *tdk^−^* strain of *B. subtilis* 168 (BKE37060), generated using ORF replacement by an erythromycin-resistant cassette, was obtained from the National BioResource Project (NIG, Japan): *B. subtilis*.^1^ Deletion of *thyA* was performed by substitution with a kanamycin resistance cassette^27^. This cassette contained the 5' and 3' flanking regions (approximately 1 kb each) of *thyA* and was prepared by gene synthesis and amplified by PCR (Supplementary Figure 2). The PCR product was directly used for transformation. Colonies resistant to both erythromycin and kanamycin were selected on solid medium. Strains with confirmed lethality at 46°C were selected from the antibiotic resistant colonies.


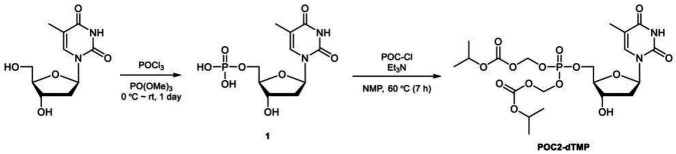



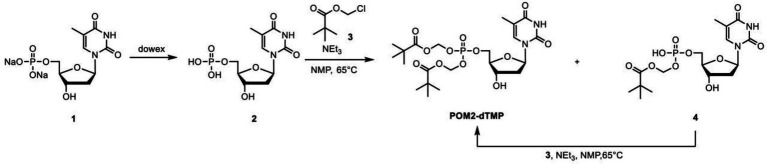


The resulting strains were subjected to PCR using genomic DNA as template to confirm substitution of *thyA* with the kanamycin resistance cassette. Genomic DNA was extracted using a DNeasy Blood & Tissue Kit (Qiagen) and amplified by nested PCR using GoTaq Green Master Mix (Promega). Primer sequences and locations are shown in Supplementary Figures 2, 5. (A,B) x (C,D) represents PCR with primers A and B followed by nested PCR with primers C and D: PCR1, (Bs-thyA-1 s,BS-KO-1as) x (Bs-thyA-2 s.Bs-KO-2as); PCR 2: (BS-KO-1 s, Bs-thyA-1as) x (BS-KO-2 s, Bs-thyA-2as); PCR 3: (Bs-thyA-1 s, Bs-thyA-1as) x (Bs-thyA-2 s, Bs-thyA-2as). PCR products were analyzed by 1% agarose gel electrophoresis.

Genome sequencing using *B. subtilis* 168 strain as a reference was used to confirm substitution of *tdk* and *thyA* with the antibiotic resistance cassettes and other off-target mutations. The integrity of the extracted DNA was checked by Genomic DNA Screen Tapes using TapeStation 2200 (Agilent) and gDNA was quantified using Quant-IT PicoGreen (Invitrogen, Grand Island, NY, United States) and a VICTOR Nivo (Perkin Elmer, Waltham, MA, United States) multimode plate reader. The sequencing libraries were prepared using a TruSeq DNA PCR-free Sample Preparation Kit (Illumina, Inc., San Diego, CA, United States). Briefly, 1 μg of genomic DNA was fragmented using adaptive focused acoustic technology (AFA; Covaris) and end-repaired to give 5'-phosphorylated, blunt-ended dsDNA. Following end-repair, DNA was size selected with a bead-based method. These DNA fragments go through addition of a single ‘A’ base and ligation of TruSeq indexing adapters. The purified libraries were quantified by qPCR based on the qPCR Quantification Protocol Guide (KAPA Library Quantification Kits for Illumina Sequencing Platforms) and qualified using a high-sensitivity DNA chip (Agilent Technologies, Waldbronn, Germany). Paired-end (2 × 150 bp) sequencing was performed by Macrogen using the NovaSeq6000 platform. Raw reads were trimmed to remove adapter sequences and low quality reads using Trimmomatic v.0.38.^2^ and read quality control was performed using FastQC v.0.11.6.^3^ Trimmed reads were mapped to the *B. subtilis* 168 reference genome (GenBank accession number AL009126.3) using BWA-MEM v0.7.17. Samtools^4^ was used to convert from SAM to BAM format and sorting of mapped sequences. The quality of the resulting BAM file was checked using Picard v.2.17.2^5^ to mark read duplicates. Variants (SNPs and small INDELs) were identified using Samtools. Variant functional annotation was performed with SnpEff v.5.0e.^6^

### Synthesis of membrane-permeable dTMP derivatives

The procedure for synthesis of POC_2_-dTMP was as follows. Thymidine (10.0 g, 41.3 mmol) was added to trimethyl phosphate (100 ml) under a nitrogen atmosphere. The resulting white suspension was cooled at 3°C, and then phosphorus oxychloride (5.75 ml, 61.9 mmol) was carefully added dropwise for 1.5 h to maintain the internal temperature below 5°C. The reaction mixture was stirred at 3°C for 6.5 h and then at room temperature for 16 h. The resulting pale yellow solution was poured into vigorously stirred ice water (1 l) and 1 M KOH aq. (250 ml) was added. The pH of this solution was >10, as checked by pH test paper. The solvent was removed under reduced pressure and the residue was purified by flash column chromatography (FCC) (ARG silica gel 200 g, acetonitrile/water: 90/10–70/30) to give the potassium salt of **1** as a pale yellow oil (35.6 g). This salt (*ca.* 13.4 g) was passed through a cation exchange resin (Dowex 50WX8-200, 100 ml). After lyophilization, the crude product **1** (10.6 g, *ca.* 15.5 mmol) was obtained as a purple oil. ^1^H NMR (270 MHz, D_2_O): *δ* 7.71 (1H), 6.32 (dd, *J* = 7.0 Hz, 1H), 4.60–4.49 (m, 1H), 4.20–4.00 (m,3H), 2.34 (dd, *J* = 7.0, 4.6 Hz, 1H), 1.88 (s, 3H).

Crude product **1** (10.6 g, *ca.* 15.5 mmol) was dissolved in 1-methyl-2-pyrrolidone (200 ml) under a nitrogen atmosphere. Triethylamine (21.1 ml, 152 mmol) was added and the mixture was stirred at 60°C for 30 min. After cooling to room temperature, the resulting pale black solution was combined with chloromethyl isopropyl carbonate (20.0 ml, 150 mmol) and the mixture was stirred at 60°C for 7 h. Brine (200 ml) was added to the resulting black solution, followed by extraction with ethyl acetate (50 ml × 9). The organic phase was dried over anhydrous sodium sulfate, filtrated to remove sodium sulfate, and evaporated at 65°C to remove 1-methyl-2-pyrrolidone to give a crude product (8.88 g). This was purified by FCC (silica gel 100 g, dichloromethane/methanol (DCM-MeOH): 99/1–97/3) to give **POC**_**2**_**-dTMP** (2.08 g, UPLC purity 79.5%) as a brown oil, followed by FCC (DIOL silica gel 120 g, DCM-MeOH: 100/0–98/2) to give **POC**_**2**_**-dTMP** (1.56 g, UPLC purity *ca.* 85%). This **POC**_**2**_**-dTMP** (1.56 g) and separately synthesized low purity **POC**_**2**_**-dTMP** (557 mg) were combined and purified by FCC (silica gel 30 g, DCM-MeOH: 99/1–97/3) and further FCC (silica gel 30 g, toluene/ethyl acetate: 80/20–20/80) to give **POC**_**2**_**-dTMP** (1.11 g, UPLC purity *ca.* 96%) as a white amorphous solid. ^1^H NMR (270 MHz, CDCl_3_): *δ* 8.65 (brs, 1H), 7.37 (s, 1H), 6.33 (dd, *J* = 6.8 Hz, 1H), 5.75–5.60 (m,4H), 5.00–4.83 (m, 2H), 4.61–4.51 (m, 1H), 4.40–4.30 (m, 2H), 4.11–4.02 (m, 1H), 3.03 (brs, 1H),2.43–2.31 (m, 1H), 2.26–2.12 (m, 1H), 1.93 (s, 3H), 1.35–1.25 (m, 12H). LCMS (ESI+): m/z calcd. For [M] + = 555.4 (Supplementary Figure 10).

The procedure for synthesizing POM_2_-dTMP was as follows: **1** (8.0 g, 22 mmol) was dissolved in ultrapure water and passed through a cation exchange resin (Dowex 50WX8-200, 130 ml). After lyophilization, **2** was obtained as a white solid (6.6 g, 20 mmol). ^1^H NMR (270 MHz, D_2_O): *δ* 7.71 (1H), 6.32 (dd, *J* = 7.0 Hz, 1H), 4.60–4.49 (m, 1H), 4.20–4.00 (m, 3H), 2.34 (dd, *J* = 7.0, 4.6 Hz, 1H), 1.88 (s, 3H). **2** (6.6 g, 20 mmol) was dissolved in 1-methyl-2-pyrrolidone (136 ml) under a nitrogen atmosphere. Triethylamine (14 ml, 100 mmol) was added and the mixture was stirred at 60°C for 30 min. After cooling to room temperature, chloromethyl pivalate (14 ml, 98 mmol) was added and the mixture was stirred at 60°C for 18 h. After cooling to room temperature, the reaction mixture was poured into water (300 ml) and extracted with ethyl acetate (200 ml x 3). The aqueous phase was concentrated under reduced pressure to about 1/4 volume. The obtained brown slurry was filtrated and concentrated under reduced pressure to give a solution including **4**. The organic phase was evaporated and dissolved in ethyl acetate/hexane 80/20 (100 ml) and washed with water (100 ml x 2). The organic phase was dried over anhydrous sodium sulfate, filtrated to remove sodium sulfate, and evaporated to give crude **POM**_**2**_**-dTMP** (crude A). The solution including **4** and triethylamine (14 ml, 100 mmol) was stirred at 60°C for 30 min under a nitrogen atmosphere. After cooling to room temperature, chloromethyl pivalate (14 ml, 98 mmol) was added to the reaction mixture and this was stirred at 60°C for 2.5 h and at room temperature for 18 h. The dark brown reaction mixture was poured into water (100 ml) and extracted with ethyl acetate (100 ml x 3). The combined organic phase was evaporated and the residue was redissolved in ethyl acetate/hexane 80/20 (100 ml) and washed with water (100 ml x 2), followed by washing with water/brine 100/10 (110 ml). The organic phase was dried over anhydrous sodium sulfate, then filtrated to remove sodium sulfate and evaporated to give crude **POM**_**2**_**-dTMP** (crude B). Crude A and B were purified by FCC (DCM-MeOH: 99/1–95/5) to give **POM**_**2**_**-dTMP** (1.7 g, 15%, UPLC purity 100%) as a white solid. ^1^H NMR (270 MHz, CDCl_3_): *δ* 9.10 (brs, 1H), 7.37 (s, 1H), 6.32 (dd, *J* = 6.8 Hz, 1H), 5.75–5.58 (m, 4H), 4.58–4.48 (m, 1H), 4.40–4.25 (m, 2H), 4.11–4.00 (m, 1H), 3.53 (d, *J* = 2.7 Hz, 1H), 2.49–2.30 (m, 1H), 2.24–2.09 (m, 1H), 1.92 (s, 3H), 1.22 (s, 9H x 2). LCMS (ESI+): m/z calcd. For [M] + = 551.7 (Supplementary Figure 11).

### Counting viable cells

Bacteria were incubated overnight at 33°C in a liquid medium containing erythromycin. To obtain bacteria in the log phase, the overnight culture was diluted to 1/100 and incubated at 37°C for 2 to 3 h (OD_590_ = 0.1–0.3). One ml of the bacterial culture diluted 50-fold was used for an experiment. After the experiment, 20 μl were collected and diluted 50-fold in an antibiotic-free medium. The 250-μl diluted culture was inoculated onto a solid medium containing erythromycin in a 9-cm diameter dish. Any experiment wares were not used for spreading sample bacterial suspension, such as bacteria spreaders, to prevent uncontrolled sample-loss due to adhering to the wares. The bacterial suspension was inoculated as several drops and the inoculated plate was tilted to spread the inoculum. After overnight incubation at 37°C, the number of colonies was counted.

### Determination of dose-effect curves

High concentration solutions of POM_2_-dTMP were prepared at 400 mM in 50% ethanol or at 800 mM in 100% DMSO. A 2- or 3-fold dilution series was prepared using the same solvent. The dilution series was diluted again to prepare a medium containing twice the final concentration. The medium was shaken at 46°C for about 2 h to dissolve the dTMP derivative completely. The medium containing the derivative was mixed with an equal volume of diluted log-growth phase bacterial suspension. To allow bacteria to uptake the dTMP derivative, the medium was incubated at 37°C for 10 min. To count the number of bacteria at time 0, an aliquot of 20 μl was collected and plated as described above. Bacterial cultures were incubated at 46°C and then plated using the same procedure. The plates were incubated overnight at 37°C and viable bacteria were detected as colonies. Rescue effects of the dTMP derivatives were evaluated by comparing viable counts before and after incubation at 46°C.

### Determination of escaper frequency

The *tdk^−^ thyA^−^* strain in the log growth phase was diluted in antibiotic-free medium to approximately OD_590_ = 0.1. Further 10-fold dilution series were prepared, from 10^−1^ to 10^−5^-fold. The diluted bacterial suspension (250 μl) was inoculated onto a solid medium containing erythromycin. After overnight incubation at 46°C, survivors were detected as colonies. The total number of inoculated bacteria was calculated from the number of colonies formed on plates incubated at 37°C. Surviving colonies were again diluted and inoculated onto two plates. To confirm that survivors were escapers carrying irreversible genetic mutations, the temperature sensitivity of the survivors was retested by incubating them separately at 37°C and 46°C.

### Growth inhibition By solvents

The *tdk^−^* strain in the log growth phase was diluted to 10^4^ c.f.u/ml using liquid medium containing erythromycin and various concentrations of ethanol or DMSO. The bacterial suspension was incubated at 46°C for 100 min and then cooled to 0°C to stop growth. After 50-fold dilution with fresh medium, The diluted bacterial suspension (250 μl) was inoculated onto a solid medium containing erythromycin. Growth inhibition was evaluated relative to growth in medium without ethanol or DMSO.

### Nucleotide quantification

Microbial cells were harvested by centrifugation at 5,800×*g* at 4°C for 5 min, after which the cells were washed twice with 10 ml of Milli-Q water. The cells were then immersed in 1,600 μl of methanol followed by ultrasonication. The cell extract was treated with 1,100 μl of Milli-Q water containing internal standards (H3304-1002, Human Metabolome Technologies, Inc. (HMT), Tsuruoka, Yamagata, Japan) and left at room temperature for 30 s, after which the mixture was cooled on ice and centrifuged at 2,300×*g* at 4°C for 5 min. Subsequently, 700 μl of supernatant was centrifugally filtered through a Millipore 5-kDa cutoff filter (Ultrafree MC-PLHCC, HMT) at 9,100×*g* at 4°C for 120 min to remove macromolecules. The filtrate was evaporated to dryness under vacuum and reconstituted in 50 μl of Milli-Q water for metabolome analysis at HMT.

Nucleotide analysis was conducted using capillary electrophoresis time-of-flight mass spectrometry (CE-TOFMS) based on methods described previously (Soga et al., 2002; Ohashi et al., 2008). Briefly, this analysis was carried out using an Agilent CE capillary electrophoresis system equipped with an Agilent 6,210 TOF mass spectrometer (Agilent Technologies, Inc., Santa Clara, CA, United States). The system was controlled by Agilent G2201AA ChemStation software ver. B.03.01 (Agilent Technologies) and connected by a fused silica capillary (50 μm *i.d.* × 80 cm total length) with commercial electrophoresis buffer (I3302-1023 for anion analyses, HMT) as the electrolyte. Areas of the target nucleotide peaks were normalized to internal standards and sample amount.

### Morphological analysis

The morphology of live bacteria was analyzed using a Nomarski differential interference contrast microscope Axioskop2 (Zeiss, Jena, Germany) equipped with a photography device CoolSnap ver. 1.1 (Roper Industries, Sarasota, FL, United States).

### Sustainable proliferation By POM_2_-dTMP supplementation

The *tdk^−^ thyA^−^* strain in the log growth phase was inoculated in 1 ml of medium containing 3 mM POM_2_-dTMP and erythromycin. To count viable bacteria at *T* = 0, an aliquot of 20 μl of bacterial culture was withdrawn, diluted in 1 ml of antibiotic-free medium, and inoculated (250 μl) onto solid medium containing erythromycin. The bacterial culture was incubated at 46°C for 30 min and then the same procedure was repeated. The culture was then mixed with an equal volume of medium containing fresh POM_2_-dTMP preheated to 46°C, aliquoted (1 ml) and recultured. Viable bacterial counts were determined before and after incubation. Recultivation was repeated once more. The total viable bacterial count was calculated from the dilution rate.

### Statistics

Statistical analyses were performed using Welch’s t-test in Excel ver.14.0.

## Data availability statement

The raw data supporting the conclusions of this article will be made available by the authors, without undue reservation.

## Author contributions

The author confirms being the sole contributor of this work and has approved it for publication.

## Funding

This work was supported by JSPS grant number 20H03158.

## Conflict of interest

The author declares that the research was conducted in the absence of any commercial or financial relationships that could be construed as a potential conflict of interest.

## Publisher’s note

All claims expressed in this article are solely those of the authors and do not necessarily represent those of their affiliated organizations, or those of the publisher, the editors and the reviewers. Any product that may be evaluated in this article, or claim that may be made by its manufacturer, is not guaranteed or endorsed by the publisher.

## Footnotes

^1^ https://shigen.nig.ac.jp/bsub/

^2^ http://www.usadellab.org/cms/?page=trimmomatic

^3^ https://www.bioinformatics.babraham.ac.uk/projects/fastqc/

^4^ http://samtools.sourceforge.net/

^5^ http://broadinstitute.github.io/picard/

^6^ https://pcingola.github.io/SnpEff/
