# Supplementary material for: A strategy for addicting transgene-free bacteria to synthetic modified metabolites
Source: Front Microbiol. 2023 Feb 10;14:1086094. doi: 10.3389/fmicb.2023.1086094 (PMC9950777; doi:10.3389/fmicb.2023.1086094)
Supplement: Supplementary file 1 [file Data_Sheet_1.docx]

**
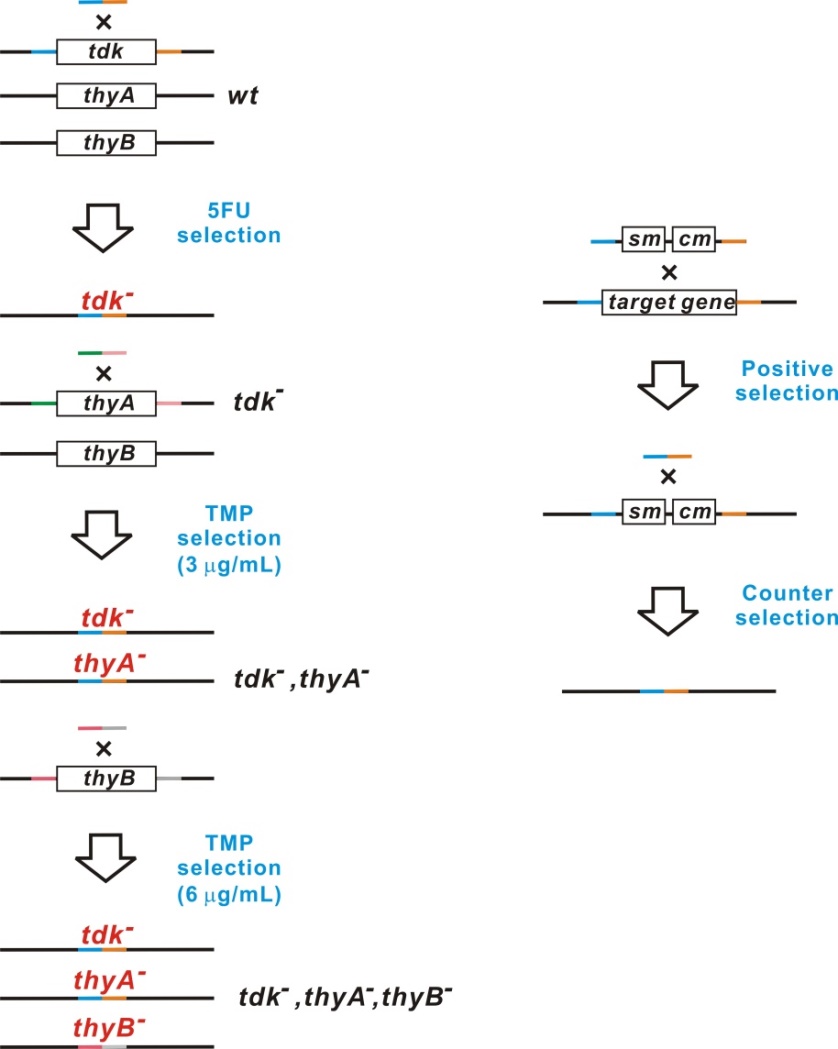
**

**Supplementary Figure 1. A possible strategy for transgene-free construction of a *B. subtilis tdk^-^ thyA^-^ thyB^-^* strain.** All gene disruptions are performed using homologous recombination with the natural competence of *B. subtilis*. Target genes are deleted by substitution with fragments of fused upstream and downstream homologous sequences. The left panel shows the selection procedure using the unique phenotypes observed in loss-of-function mutants (García-González et al., 2017; Hosseini et al., 2018). The right panel shows a two-step selection-based genome editing method that does not use gene-specific phenotypes by substitutions and deletions with gene cassettes containing a positive selection marker, such as antibiotic resistance genes, and a counter-selection marker, such as conditionally toxic genes (Zhang et al., 2006). The intrinsic phenotype-independent method can be used to delete any genes. *wt*, wild-type. 5FU, 5-fluorouracil. TMP, trimethoprim. Sm, selection marker. Cm, counter-selection marker.

**1 tgtcgcaatc ataatgcttc ttatcattgt cgtaatactt tttgtaatat gtttttttcc**

**thyA-KO-s　→**

**61 aagtataata ctcttcttta tattttttat aataacccaa ttaaattcct cctttatgtt**

**121 attcgaattt aatttatgaa gtttaaatga tatggtatgg tcaaatgaaa taagaatagt**

**181 aaaaagaggc tgtttaaaac actttctgaa aaacaaaacg gatagctgtt tatgaaaata**

**241 agagcgagag agagcactca atcactgtaa aataatagta aagtgtatag agagattagt**

**301 atacgtaaaa agggactttc tcattgaaga atatttgaac gttatggtta agattcaaga**

**361 gctctatgat gtgatttttc acactgcttt tgaaggattt agagttagac atcaaaaaca**

**421 aacagcaaaa ccggctagaa acagatcata taaggaatga accgctgcca aatatcataa**

**481 aaaagttgtt aatgatcaaa tgaatgaaat tagagagaat ttattttaaa gaaagcccaa**

**541 ttgcacatgg acaaatgaca attgatattg aggtaaaata caatgcatta aatcagaaaa**

**601 agctattgga ggatttaatg tgtttagaca atttccaatt tggtatacac aaacacctga**

**661 ctatttgaat ttttatgtac cgcaatatca aaccatttcg tataatcctc aacaatgtta**

**721 tcaacggtgt atgtaccaaa ctggcggtaa ctatgagcta tgtgacagac tatgttatgg**

**781 agaaatacag gtgtaaaaga gggggattaa ctcctcttta aacacacagt gagtggaata**

**841 agatcctcac tttatctgca agtgcttagt atttgcgata atattgcatt cgtaataaat**

**901 tatgcttagc aactgaaaat gaaagaagga tatgaatagt catgGCAGGC GAGAAAGGAG**

**961 AGAGAACGCA AGGAGAGGCA CGCGAGGGAG GAAAGGCAGG ATACCGTTCG TATAGCATAC**

**1021 ATTATACGAA GTTATGAATT CGTTTGATTT TTAATGGATA ATGTGATATA ATCTTTAAAT**

**1081 ACTGTAGAAA AGAGGAAGGA AATAATAAAT GGCTAAAATG AGAATATCAC CGGAATTGAA**

**←　Bs-KO-2as**

**1141 AAAACTGATC GAAAAATACC GCTGCGTAAA AGATACGGAA GGAATGTCTC CTGCTAAGGT**

**←　Bs-KO-1as**

**1201 ATATAAGCTG GTGGGAGAAA ATGAAAACCT ATATTTAAAA ATGACGGACA GCCGGTATAA**

**1261 AGGGACCACC TATGATGTGG AACGGGAAAA GGACATGATG CTATGGCTGG AAGGAAAGCT**

**1321 GCCTGTTCCA AAGGTCCTGC ACTTTGAACG GCATGATGGC TGGAGCAATC TGCTCATGAG**

**1381 TGAGGCCGAT GGCGTCCTTT GCTCGGAAGA GTATGAAGAT GAACAAAGCC CTGAAAAGAT**

**1441 TATCGAGCTG TATGCGGAGT GCATCAGGCT CTTTCACTCC ATCGACATAT CGGATTGTCC**

**1501 CTATACGAAT AGCTTAGACA GCCGCTTAGC CGAATTGGAT TACTTACTGA ATAACGATCT**

**1561 GGCCGATGTG GATTGCGAAA ACTGGGAAGA AGACACTCCA TTTAAAGATC CGCGCGAGCT**

**1621 GTATGATTTT TTAAAGACGG AAAAGCCCGA AGAGGAACTT GTCTTTTCCC ACGGCGACCT**

**1681 GGGAGACAGC AACATCTTTG TGAAAGATGG CAAAGTAAGT GGCTTTATTG ATCTTGGGAG**

**1741 AAGCGGCAGG GCGGACAAGT GGTATGACAT TGCCTTCTGC GTCCGGTCGA TCAGGGAGGA**

**1801 TATCGGGGAA GAACAGTATG TCGAGCTATT TTTTGACTTA CTGGGGATCA AGCCTGATTG**

**Bs-KO-1s　→　　　　　　　Bs-KO-2s　→**

**1861 GGAGAAAATA AAATATTATA TTTTACTGGA TGAATTGTTT TAGTACCTAG ACTCGAGATA**

**1921 ACTTCGTATA GCATACATTA TACGAACGGT AGAGAGAGCA CAGATACGGC GACGACACCG**

**1981 AAGCAGAGCG AAGCAGTGAC AGGAGCCTCG taatgctgcc tttttattgt gcagtgaata**

**2041 gatagcaggt atcctaattt cattaagcaa tctggaagat gaataaaaat tgaaggacaa**

**2101 acacgtataa tacataaaaa agattaactc tacagttaat cttttttatt cagaagaaaa**

**2161 tatcctaact ttgaaactaa atacaaagta aaagcaatca ttacagttct agatattaca**

**2221 attccatgaa tagctagatc atatccagca ggtatcaacg catttgtatt acacataaaa**

**2281 tatatagata ttagaagtgc tacaataact aaaatcattc caaaaagact tgttttttca**

**2341 tatttcatac caatttccac ccttattaaa gttaggttta aacaaaagag ctgaagaaac**

**2401 gaactatgac cagtatgctc caaggaaaac cgccagacaa tgctggcggc tttttgctgc**

**2461 ttcgtttatt tattaacaga gatcgtaacg ttatttcctg caactgaaac ctttgcgaaa**

**2521 tcctgtgtat ctaccacagg agtgacggct ttattgtcga tagtgatgtt gttaaaagag**

**2581 gtcgaaaagg ttgctttgat cgcatcacgc tggctttcat acgaaacagc cgcggtagca**

**2641 agtgttttcc attttgaaat cgtgccgatg ttggttcctt gaatttctgt gataatccgt**

**2701 ccggtgtagc cgtcattgtt cgttccccaa agggtcatgc gcgtattgcc gcttaaattt**

**2761 ttatagattg tcatttggac tgtgcttcca gggcggaagc ctttattgta tgtgaatttg**

**2821 tcttttcctt cgatgtacgt ttcttcattt ttggacccaa ccttcatgag gggcttccag**

**2881 acgttgtatt gtttgctgta ctgaagcccg atatcggctt cagttccgct tgttgcccta**

**2941 aagccagaat aaatataggg agtagagacc gagtcgttgt ttttgacagt ttcaggaagc**

**3001 acgattttag ctg**

**←　thyA-KO-as**

**Supplementary Figure 2. Nucleotide sequence of the *thyA* knockout cassette.** The knockout cassette was prepared by gene synthesis. The full length was amplified by PCR using thyA-KO-s and thyA-KO-as primers and used directly to delete *thyA* in the *B. subtilis* 168 *tdk^-^* strain using homologous recombination. Sequences homologous to upstream (1-941) and downstream (2011-3013) of *thyA* are indicated by lower case letters. Translation initiation and termination codons for *thyA* are highlighted in magenta. ORFs for the kanamycin resistance gene are highlighted in green.

**
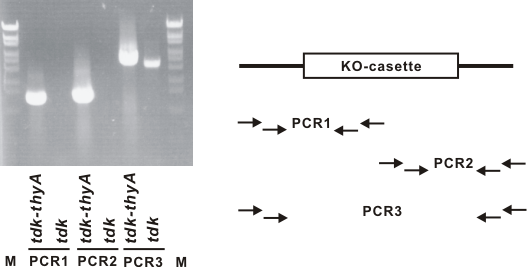
**

**Supplementary Figure 3. PCR confirmation of *thyA* deletion.** In *tdk^-^ thyA^-^*, substitution of *thyA* with the knockout cassette, shown in Supplementary Figure 2, was confirmed by three different PCRs, PCR1-PCR3, using genomic DNA as template. The parental strain, *tdk^-^*, was used as a control. Primer sequences and locations are shown in Supplementary Figures 2 and 5. The predicted size of PCR products for *tdk^-^ thyA^-^* (*tdk^-^*) are 1,233 (none), 1,294 (none) and 3,232 (3,000) for PCR1, PCR2 and PCR3, respectively. M, molecular mass marker (*lambda/*StyI; 19.33, 7.74, 6.22, 4.26, 3.47, 2.69, 1.88, 1.49 and 0.93 kbp).

**
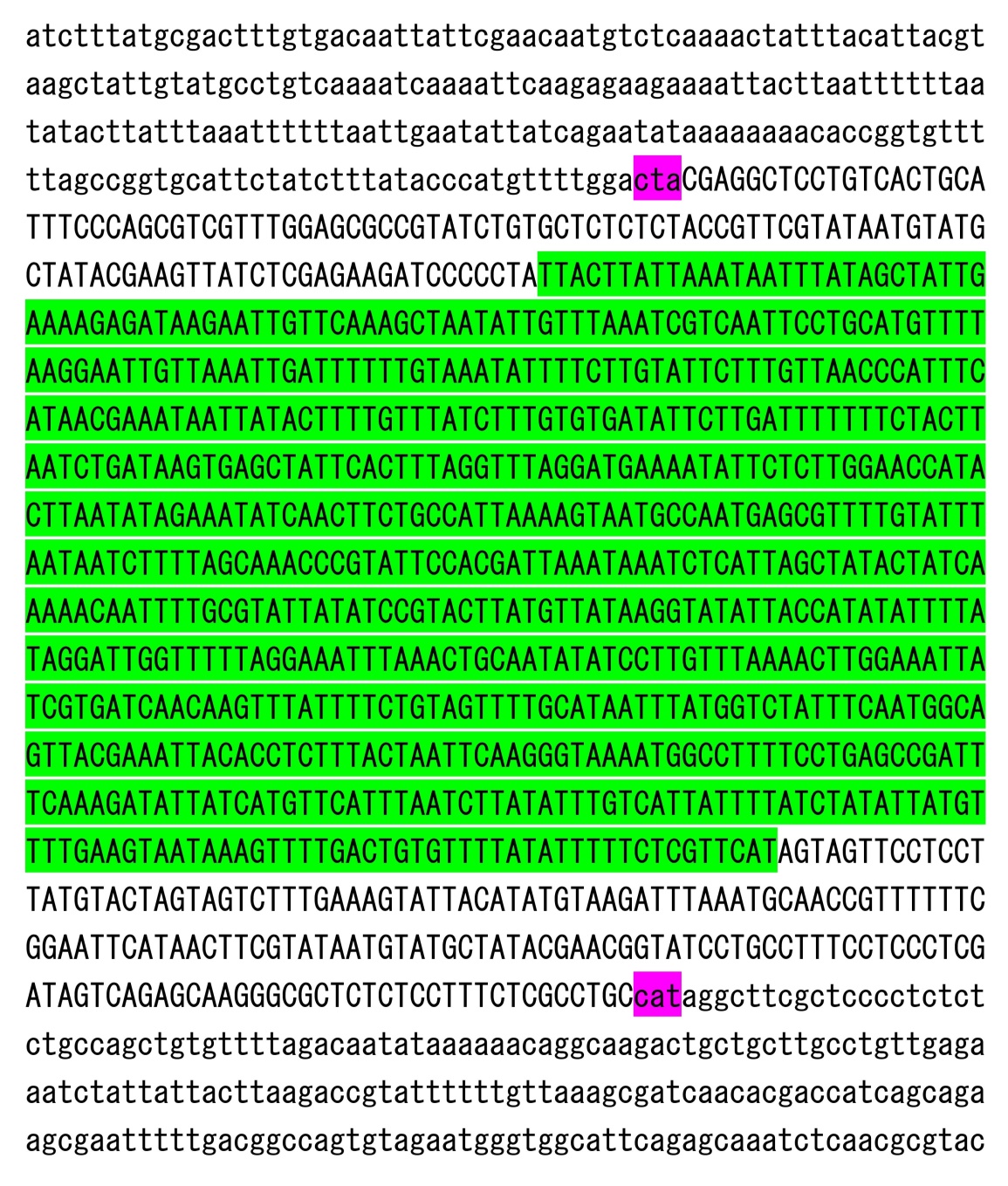
**

**Supplementary Figure 4. Nucleotide sequence of the *tdk* locus in the *tdk^-^ thyA^-^* strain.** The entire genome sequence of the *tdk^-^ thyA^-^* strain was analyzed by Illumina sequencing. The sequence near the locus of *tdk* is shown. The sequence between the translation start and stop codons of *tdk* (highlighted in magenta) was substituted with the *tdk* knockout cassette containing the erythromycin resistance gene (highlighted in green).

**aaagcccccggtatgtcgccagggaataaatagacgtttaagagatttagtgatgttttt**

**Bs-thyA-1s →**

**tatatgattcgacaaaatcataataatgatcatcgtcatcataatactttttttcgtaat**

**gataatcgtgatcatcgtaatactttttatcatggtcatcatactttttgtcgtaatcat**

**Bs-thyA-2s →**

**aatgttttttatcatgatcataatacttgtcgcaatcataatgcttcttatcattgtcgt**

**aatactttttgtaatatgtttttttccaagtataatactcttctttatattttttataat**

**aacccaattaaattcctcctttatgttattcgaatttaatttatgaagtttaaatgatat**

**ggtatggtcaaatgaaataagaatagtaaaaagaggctgtttaaaacactttctgaaaaa**

**caaaacggatagctgtttatgaaaataagagcgagagagagcactcaatcactgtaaaat**

**aatagtaaagtgtatagagagattagtatacgtaaaaagggactttctcattgaagaata**

**tttgaacgttatggttaagattcaagagctctatgatgtgatttttcacactgcttttga**

**aggatttagagttagacatcaaaaacaaacagcaaaaccggctagaaacagatcatataa**

**ggaatgaaccgctgccaaatatcataaaaaagttgttaatgatcaaatgaatgaaattag**

**agagaatttattttaaagaaagcccaattgcacatggacaaatgacaattgatattgagg**

**taaaatacaatgcattaaatcagaaaaagctattggaggatttaatgtgtttagacaatt**

**tccaatttggtatacacaaacacctgactatttgaatttttatgtaccgcaatatcaaac**

**catttcgtataatcctcaacaatgttatcaacggtgtatgtaccaaactggcggtaacta**

**tgagctatgtgacagactatgttatggagaaatacaggtgtaaaagagggggattaactc**

**ctctttaaacacacagtgagtggaataagatcctcactttatctgcaagtgcttagtatt**

**tgcgataatattgcattcgtaataaattatgcttagcaactgaaaatgaaagaaggatat**

**gaatagtcatgGCAGGCGAGAAAGGAGAGAGAACGCAAGGAGAGGCACGCGAGGGAGGAA**

**AGGCAGGATACCGTTCGTATAGCATACATTATACGAAGTTATGAATTCGTTTGATTTTTA**

**ATGGATAATGTGATATAATCTTTAAATACTGTAGAAAAGAGGAAGGAAATAATAAATGGC**

**TAAAATGAGAATATCACCGGAATTGAAAAAACTGATCGAAAAATACCGCTGCGTAAAAGA**

**TACGGAAGGAATGTCTCCTGCTAAGGTATATAAGCTGGTGGGAGAAAATGAAAACCTATA**

**TTTAAAAATGACGGACAGCCGGTATAAAGGGACCACCTATGATGTGGAACGGGAAAAGGA**

**CATGATGCTATGGCTGGAAGGAAAGCTGCCTGTTCCAAAGGTCCTGCACTTTGAACGGCA**

**TGATGGCTGGAGCAATCTGCTCATGAGTGAGGCCGATGGCGTCCTTTGCTCGGAAGAGTA**

**TGAAGATGAACAAAGCCCTGAAAAGATTATCGAGCTGTATGCGGAGTGCATCAGGCTCTT**

**TCACTCCATCGACATATCGGATTGTCCCTATACGAATAGCTTAGACAGCCGCTTAGCCGA**

**ATTGGATTACTTACTGAATAACGATCTGGCCGATGTGGATTGCGAAAACTGGGAAGAAGA**

**CACTCCATTTAAAGATCCGCGCGAGCTGTATGATTTTTTAAAGACGGAAAAGCCCGAAGA**

**GGAACTTGTCTTTTCCCACGGCGACCTGGGAGACAGCAACATCTTTGTGAAAGATGGCAA**

**AGTAAGTGGCTTTATTGATCTTGGGAGAAGCGGCAGGGCGGACAAGTGGTATGACATTGC**

**CTTCTGCGTCCGGTCGATCAGGGAGGATATCGGGGAAGAACAGTATGTCGAGCTATTTTT**

**TGACTTACTGGGGATCAAGCCTGATTGGGAGAAAATAAAATATTATATTTTACTGGATGA**

**ATTGTTTTAGTACCTAGACTCGAGATAACTTCGTATAGCATACATTATACGAACGGTAGA**

**GAGAGCACAGATACGGCGACGACACCGAAGCAGAGCGAAGCAGTGACAGGAGCCTCGtaa**

**tgctgcctttttattgtgcagtgaatagatagcaggtatcctaatttcattaagcaatct**

**ggaagatgaataaaaattgaaggacaaacacgtataatacataaaaaagattaactctac**

**agttaatcttttttattcagaagaaaatatcctaactttgaaactaaatacaaagtaaaa**

**gcaatcattacagttctagatattacaattccatgaatagctagatcatatccagcaggt**

**atcaacgcatttgtattacacataaaatatatagatattagaagtgctacaataactaaa**

**atcattccaaaaagacttgttttttcatatttcataccaatttccacccttattaaagtt**

**aggtttaaacaaaagagctgaagaaacgaactatgaccagtatgctccaaggaaaaccgc**

**cagacaatgctggcggctttttgctgcttcgtttatttattaacagagatcgtaacgtta**

**tttcctgcaactgaaacctttgcgaaatcctgtgtatctaccacaggagtgacggcttta**

**ttgtcgatagtgatgttgttaaaagaggtcgaaaaggttgctttgatcgcatcacgctgg**

**ctttcatacgaaacagccgcggtagcaagtgttttccattttgaaatcgtgccgatgttg**

**gttccttgaatttctgtgataatccgtccggtgtagccgtcattgttcgttccccaaagg**

**gtcatgcgcgtattgccgcttaaatttttatagattgtcatttggactgtgcttccaggg**

**cggaagcctttattgtatgtgaatttgtcttttccttcgatgtacgtttcttcatttttg**

**gacccaaccttcatgaggggcttccagacgttgtattgtttgctgtactgaagcccgata**

**tcggcttcagttccgcttgttgccctaaagccagaataaatatagggagtagagaccgag**

**tcgttgtttttgacagtttcaggaagcacgattttagctgtaaaaacggcgcccgtactg**

**ttcaggtagacacggccgccgattccgttaggcagctcggatgctgcttttgcgacttga**

**gcgtctgccgcgggtaaacctggtatacctgatgaaagggttccgaaaagaactgctgtt**

**←　Bs-thyA-2as**

**cctactgcacctgcaatcaatacttttttcaccaaaattcccccttttgatagtggtttc**

**←　Bs-thyA-1as**

**gccatttattataccttattttggaactggatttacgtatataaatcgacaaaatatttc**

**Supplementary Figure 5. Nucleotide sequence of the *thyA* locus in the *tdk^-^ thyA^-^* strain.** The sequence near the locus of *thyA* is shown. The sequence between the translation start and stop codons of *thyA* (highlighted in magenta) was substituted with the *thyA* knockout cassette containing the kanamycin resistance gene (highlighted in green) shown in Supplementary Figure 2.

**
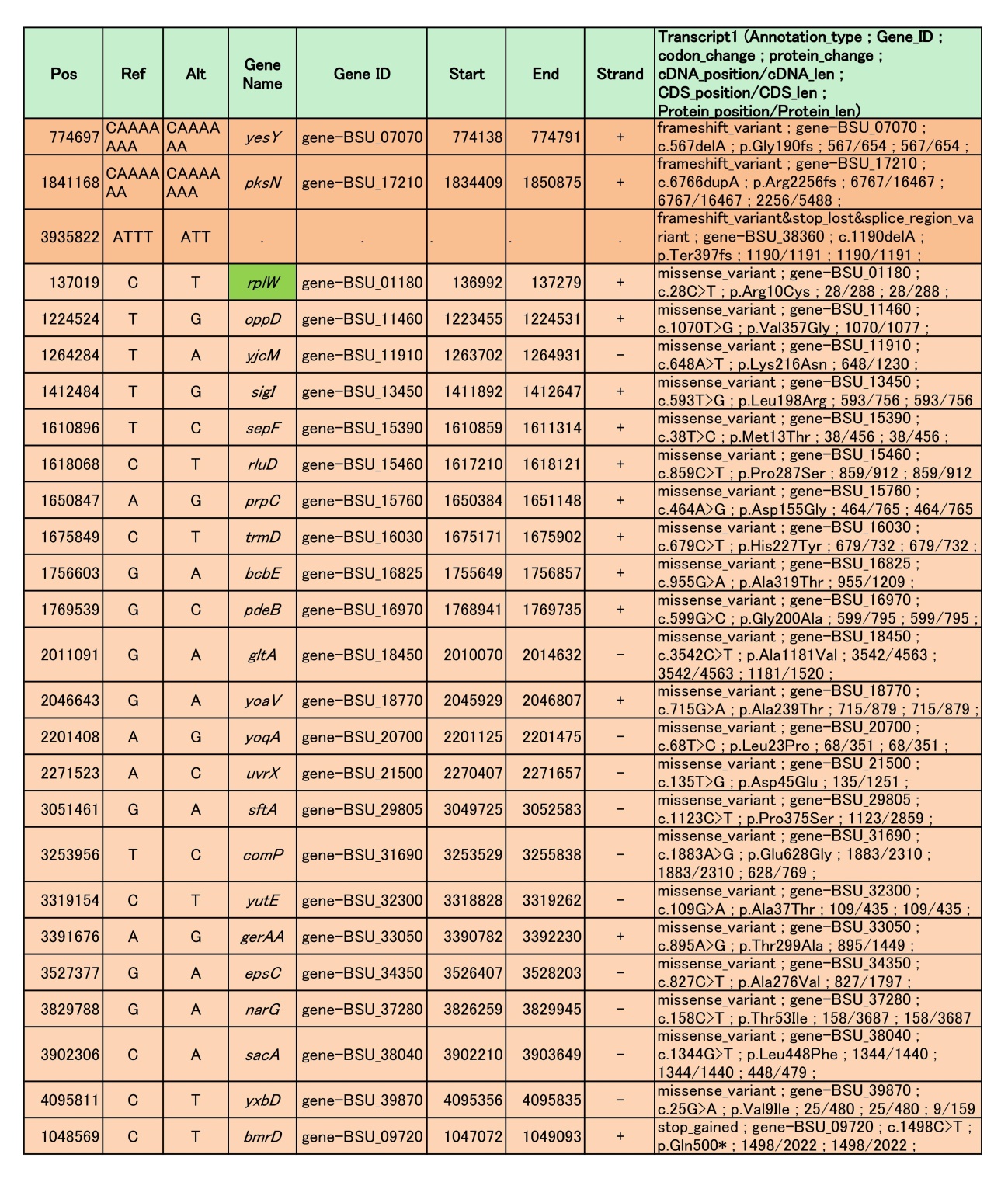
**

**Supplementary Figure 6. Mutations detected in the *tdk^-^ thyA^-^* strain.** Unintentional mutations detected in genome sequencing of the *tdk^-^ thyA^-^* strain that differ from those in the parental *B. subtilis* 168 genome are listed. The essential gene (*rplW*) is highlighted in green.

**
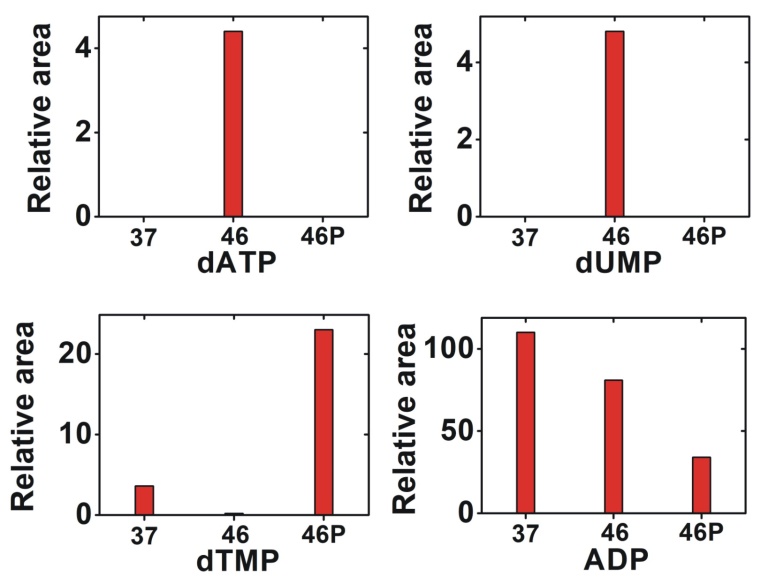
**

**Supplementary Figure 7. Nucleotide metabolism in *tdk^-^ thyA^-^*.** The *tdk^-^ thyA^-^* strain was incubated at 37°C in the absence of POM_2_-dTMP, and at 46°C in the absence and presence of 3 mM POM_2_-dTMP for 30 min. Nucleotide quantification results are shown as 37, 46, and 46P, respectively. Quantification was performed for three deoxynucleotides, dATP, dUMP, and dTMP, which change markedly upon thymineless death due to loss of Thy function. ADP was also examined as a control. This is a single-trial experiment.

**A**

**
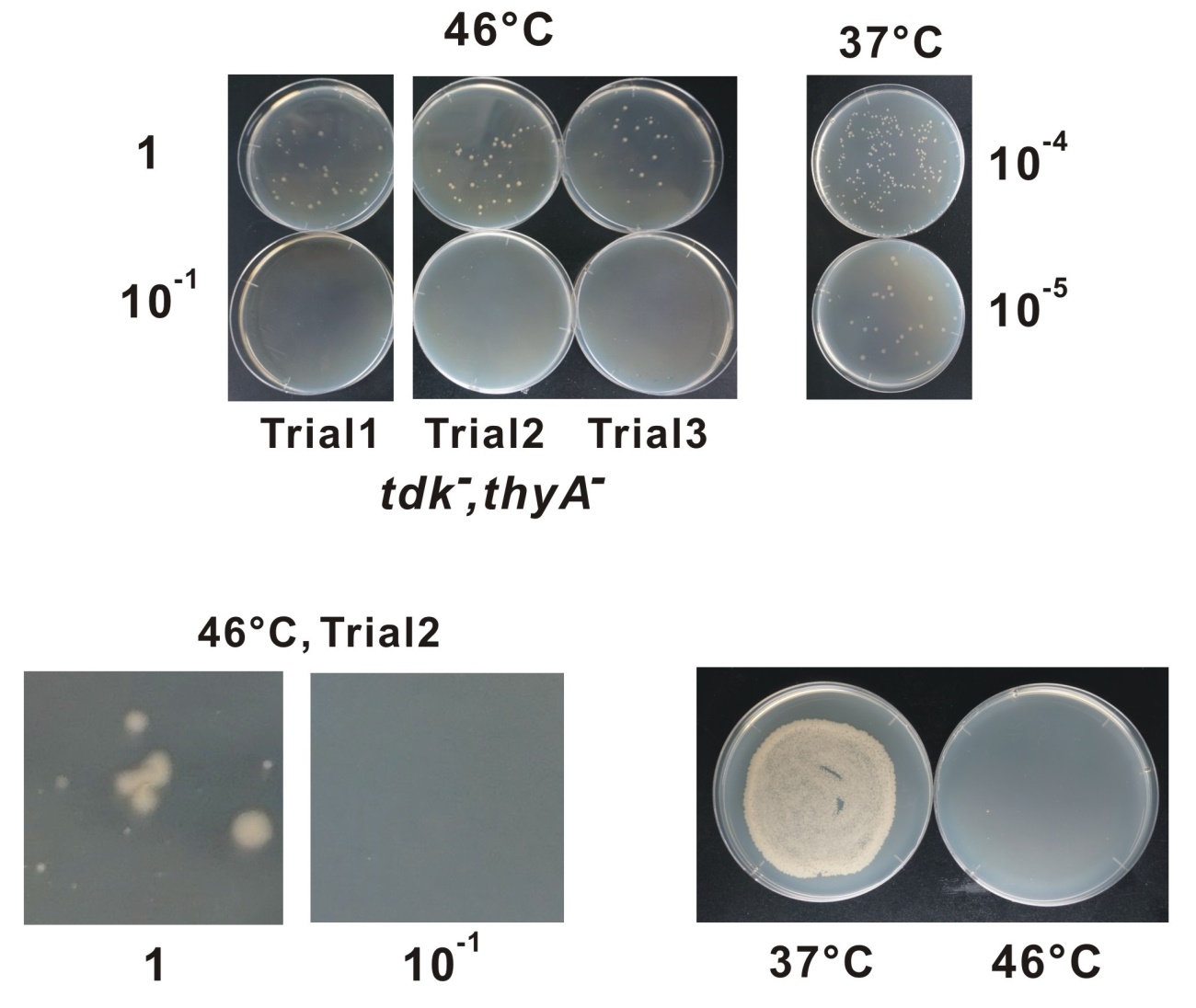
**

**B**

**C**

**Supplementary Figure 8. Detection of pseudo-escapers in high density inoculation. A** Penetration of lethality in *tdk^-^ thyA^-^* double mutant bacteria at 46°C. A 10-fold dilution series of the *tdk^-^ thyA^-^* strain (OD_590_ = 0.3) in the log growth phase was prepared and inoculated at 250 μL on each plate. In the left panel, plates were incubated overnight at 46°C. When undiluted bacterial cultures containing 10^7^ cells/plate were inoculated, many colonies were detected. However, no colonies were detected at 10-fold dilution or higher containing <10^6^ cells/plate, even though a number of colonies was predicted to appear based on the dilution factor. The right panel shows plates incubated at 37°C to measure the total number of inoculated bacteria. **B** Magnified image of colonies. Dilution ratios are shown below. **C** Retest for temperature sensitivity of survivors. Colonies obtained from plates inoculated with undiluted bacterial cultures were suspended, reinoculated on plates, and incubated at 37°C and 46°C. A typical example is shown. Uneven colony distribution is an artificial phenomenon caused by uneven inoculation.

**A**

**
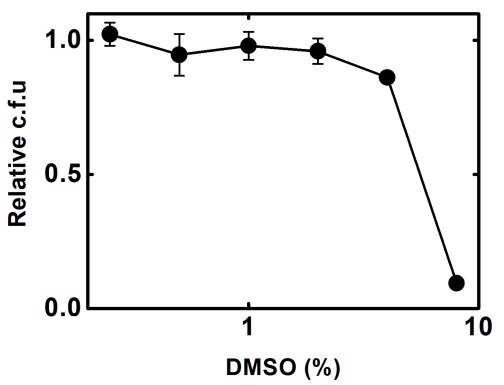

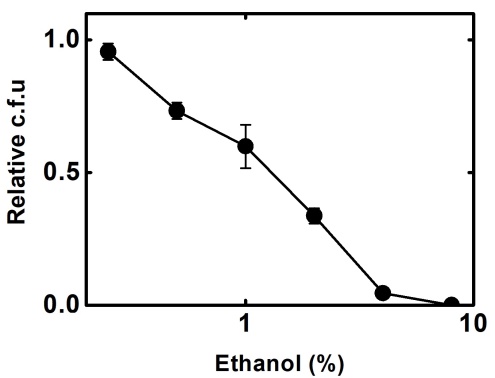
**

**
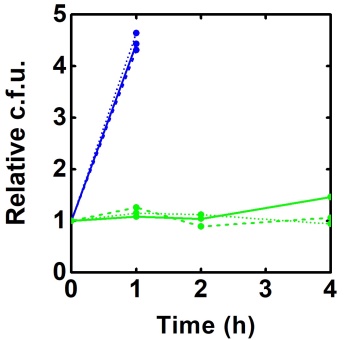
B**

**
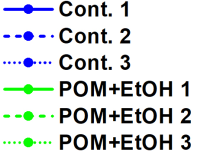
**

**Supplementary Figure 9. Growth inhibition by high concentrations of ethanol and dimethylsulfoxide. A** Dose-effect curves. The *tdk^-^* strain in the log growth phase was incubated for 100 min at 46°C in liquid medium containing various concentrations of ethanol or DMSO. Bacterial growth was detected by an increase in the number of colonies. The growth was evaluated relative to a value of 1 in the absence of ethanol or DMSO. Values are mean ± sd　of three biological replicates. **B** Growth arrest caused by POM_2_-dTMP with ethanol. Bacteria were incubated at 46°C. The number of viable bacteria was estimated at each time points, and represented a relative value (T0 = 1). Green and blue lines indicate bacterial growth in the presence and absence of 4 mM POM_2_-dTMP and 2% ethanol, respectively.

**A**

**
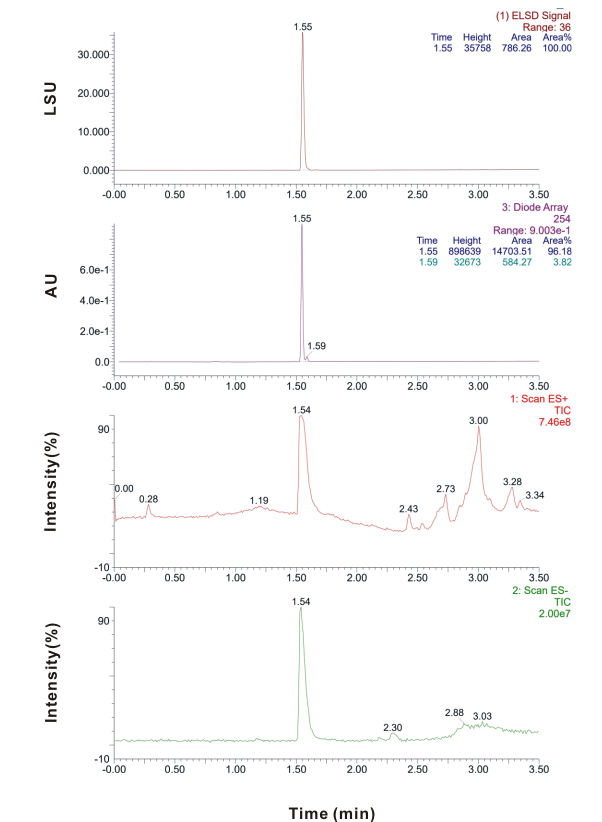
**

**B**

**
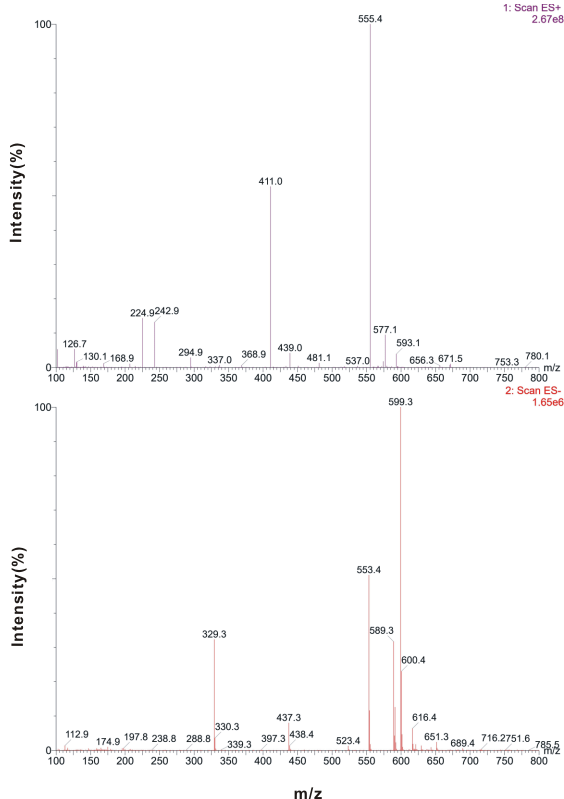
**

**C**

**
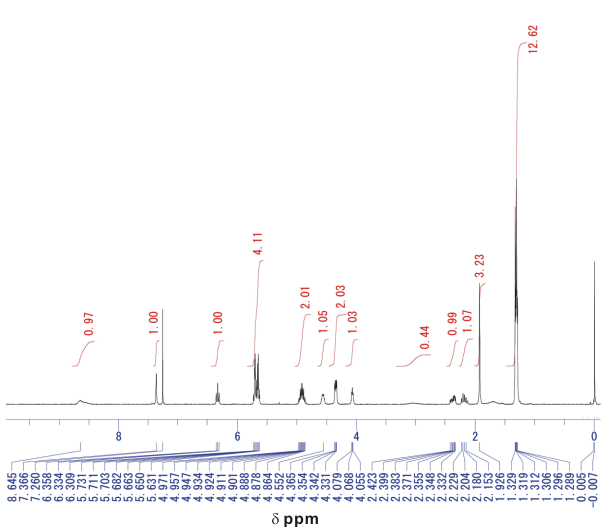
**

**Supplementary Figure 10.　Preparation of POC_2_-dTMP. A** Flash column chromatogram. Red triangle indicates POC_2_-dTMP. ESI+ and ESI-, positive and negative electrospray ionization mass spectrometry. TIC, Total ion chromatogram. **B** LC/MS ESI+ spectrum. m/z calculated for [M]+ is 555.4. **C** ^1^H NMR spectrum. See M&M for more details.

**A**

**
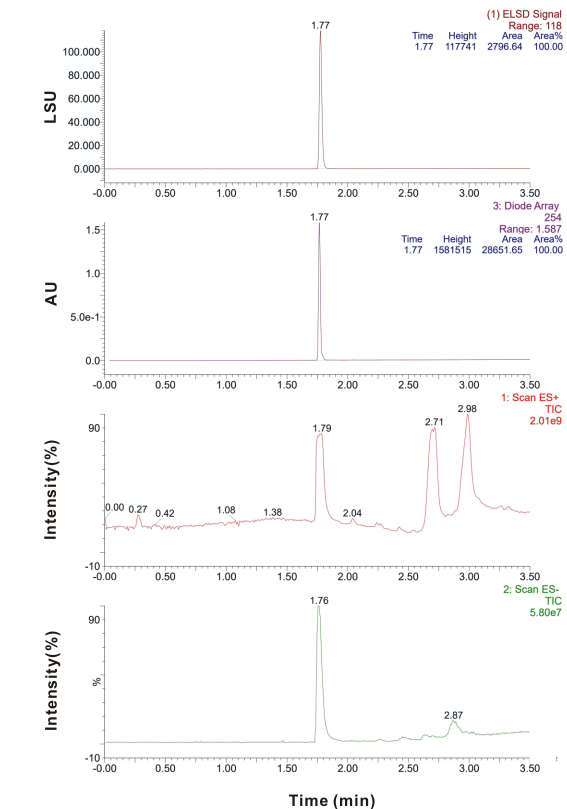
**

**B**

**
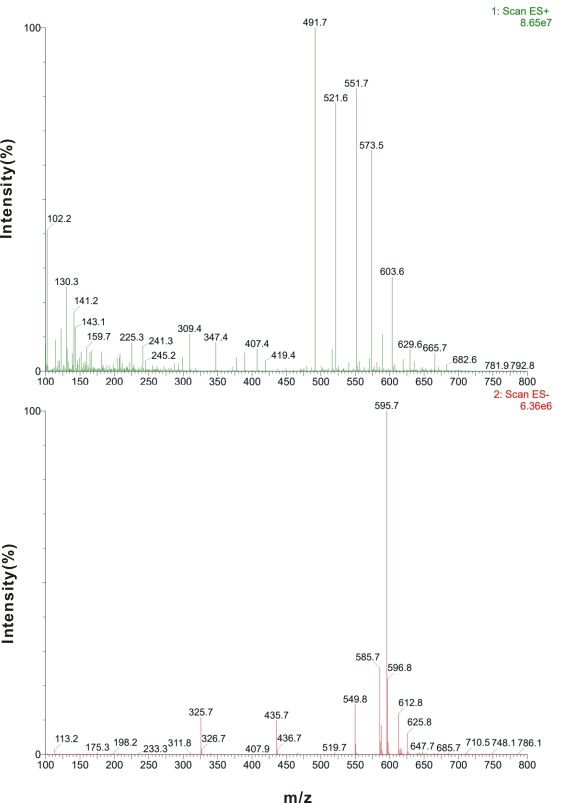
**

**C**

**
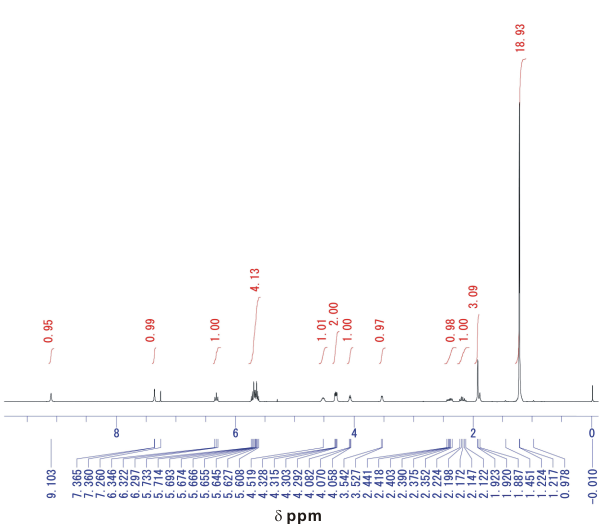
**

**Supplementary Figure 11.　Preparation of POM_2_-dTMP. A** Flash column chromatogram. Red triangle indicates POM_2_-dTMP. **B** LC/MS ESI+ spectrum. m/z calculated for [M]+ is 551.7. **C** ^1^H NMR spectrum. See M&M for more details.

**
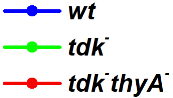

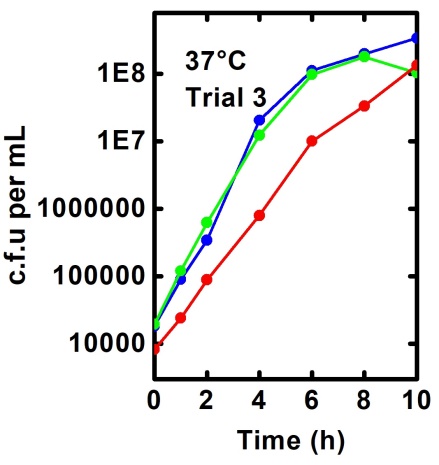

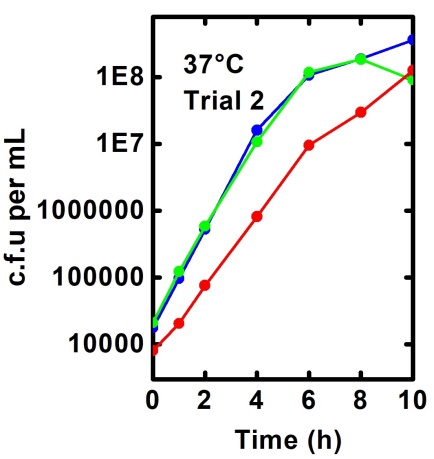

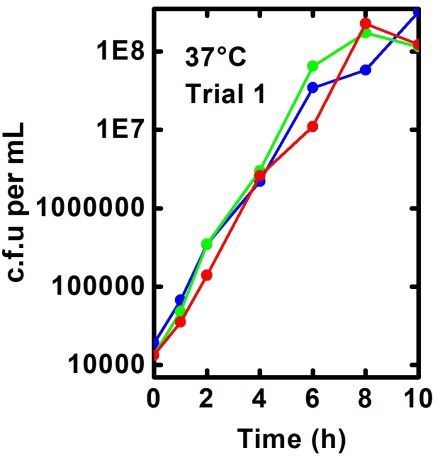
**

**
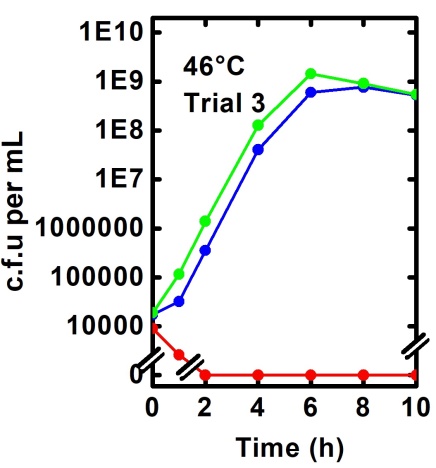

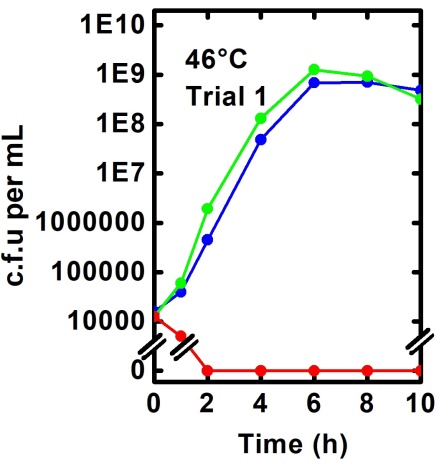

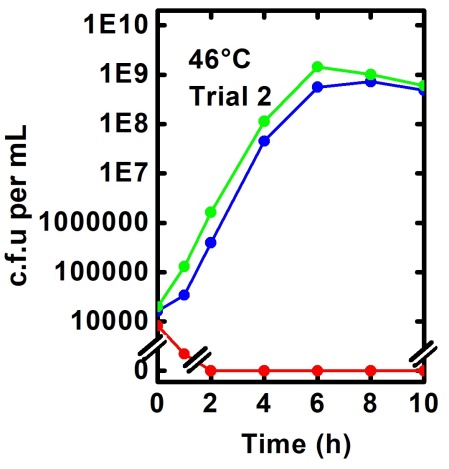
**

**Supplementary Figure 12.** **Long-term** **growth curve**. Red, green and blue lines indicate *tdk^-^ thyA^-^*, *tdk^-^* and wild-type growth, respectively.

**Supplementary References**

García-González, A. P., Ritter, A. D., Shrestha, S., Andersen, E. C., Yilmaz, L. S., Walhout, A. J. M. (2017) Bacterial metabolism affects the *C. elegans* response to cancer chemotherapeutics. *Cell* 169, 431-441.e8. Doi: 10.1016/j.cell.2017.03.046

Hosseini, S., Curilovs, A., and Cutting, S. M. (2018) Biological containment of genetically modified *Bacillus subtilis*. *Appl. Environ. Microbiol.* 84, e02334-17. Doi: 10.1128/AEM.02334-17

Zhang, X.-Z., Yan, X., Cui, Z.-L., Hong, Q., and Li, S.-P. (2006) *mazF*, a novel counter-selectable marker for unmarked chromosomal manipulation in *Bacillus subtilis. Nucleic Acids Res* 34, e71. Doi: 10.1093/nar/gkl358
